# Idiopathic remitting seronegative symmetrical synovitis with pitting edema syndrome mimicking symptoms of polymyalgia rheumatica: a case report

**DOI:** 10.1186/s13256-022-03535-z

**Published:** 2022-08-27

**Authors:** Katarzyna Tarasiuk-Stanislawek, Alexandre Dumusc, Bernard Favrat, Ioannis Kokkinakis

**Affiliations:** 1 Center for Primary Care and Public Health (Unisanté), University Center of General Medicine and Public Health, University of Lausanne, Rue du Bugnon 44, 1011 Lausanne, Switzerland; 2 Rheumatology Department, Lausanne University Hospital (CHUV), Lausanne, Switzerland

**Keywords:** RS3PE, Idiopathic remitting seronegative symmetrical synovitis pitting edema syndrome, PMR, Seronegative arthritis, Case report

## Abstract

**Background:**

Remitting seronegative symmetrical synovitis with pitting edema is a rare rheumatic condition of the elderly population that is well described but whose mechanisms remain little studied. This syndrome is characterized by symmetrical swelling located mainly on the dorsal part of the hands and the feet. Because of possible heterogeneous clinical presentation, it can easily mimic the onset of other rheumatic diseases or appear associated with them. Here we report a case of a patient who developed remitting seronegative symmetrical synovitis with pitting edema with preexisting shoulder and hip girdle pain associated with progressive fatigue, indicating a possible differential diagnosis of polymyalgia rheumatica. We reviewed and compared classification for remitting seronegative symmetrical synovitis with pitting edema and polymyalgia rheumatica and discussed other differential diagnoses.

**Case presentation:**

An 84-year-old Caucasian woman presented to our General Medicine Unit with acute onset of symmetrical hands and feet edema, leading to functional limitation due to pain and stiffness. Additionally, she was complaining about neck, shoulder, and pelvic girdle pain present for about 2 months associated with worsening asthenia. Blood tests showed an elevated level of C-reactive protein and erythrocyte sedimentation rate, as well as absence of anti-cyclic citrullinated peptide antibodies and rheumatoid factor. As all criteria of remitting seronegative symmetrical synovitis with pitting edema syndrome were present, the patient was treated with low-dose prednisone, with a rapid and complete resolution of symptoms. She remains asymptomatic without treatment 2 years after the onset of symptoms, without any evident oncologic etiology.

**Conclusions:**

This case is an example of a classic representation of remitting seronegative symmetrical synovitis with pitting edema syndrome with clinical elements suggesting a concomitant existing early stage of polymyalgia rheumatica. These two entities, classified in the group of seronegative arthritis, can coexist (up to 10% of cases), with remitting seronegative symmetrical synovitis with pitting edema appearing as an initial or late manifestation of polymyalgia rheumatica. It is essential to remind that remitting seronegative symmetrical synovitis with pitting edema is associated with a higher risk of cancer (30%). A proper diagnosis allows the clinician to precisely define the appropriate therapy duration to limit its side effects in the elderly and remain aware of the potential risk of underlying malignancy.

## Background

Remitting seronegative symmetrical synovitis with pitting edema syndrome (RS3PE) is an uncommon rheumatological entity belonging to the group of seronegative arthritis typically found among the elderly population [1–5], affecting predominantly men (2:1) [1–3, 5]. It is characterized by acute symmetric tenosynovitis and pitting edema of the dorsal part of hands and feet, leading to important functional limitation [1, 5]. Inflammatory markers are usually elevated, and rheumatoid factor (RF) and anti-cyclic citrullinated peptides (anti-CCP) antibodies are negative [1, 3–5]. After low-dose glucocorticoids, all symptoms resolve without residual sequelae [1–6]. The heterogeneous clinical manifestation of RS3PE can delay diagnosis owing to overlapping symptoms with other rheumatological diseases [2–4]. We discuss here a case of RS3PE associated with progressive asthenia, shoulder, and hip girdle pain, which can mimic clinical manifestations of polymyalgia rheumatica (PMR) [1, 2, 4–7]. It is essential to remind that RS3PE can coexist with PMR, and in such cases, it is associated with an underlying neoplastic condition in about 28% of patients [7].

## Case presentation

**Fig. 1:**
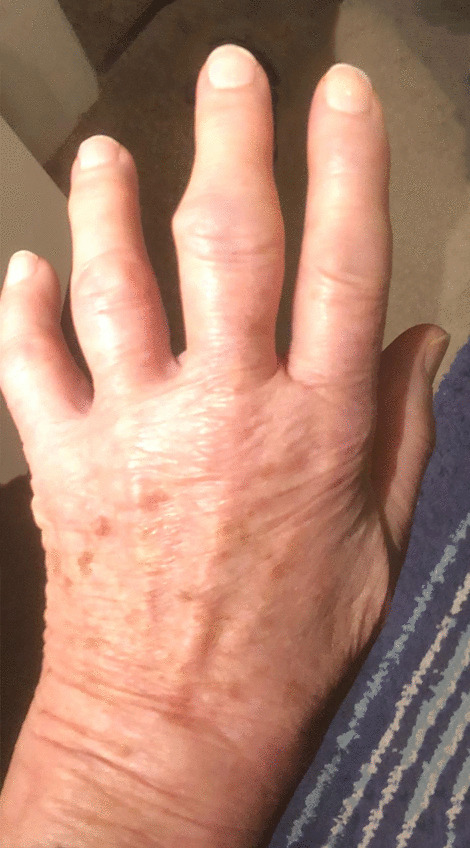
Initial clinical presentation of edema. Edema of the left hand 10 days after onset of symptoms. Deformation of the proximal interphalangeal joint of the left middle finger is due to prior existing osteoarthritis

An 84-year-old Caucasian woman, in good health, without any medication, independent in activities and instrumental activities of daily living, walking without any assisting devices, presented to our General Medicine Unit with painful swelling of the hands and the feet. She described stiffness in her hands and feet that developed abruptly 10 days before. Subsequently, she noted swelling of her hands and feet associated with significant pain and functional limitation (Fig. 1).

**Fig. 2:**
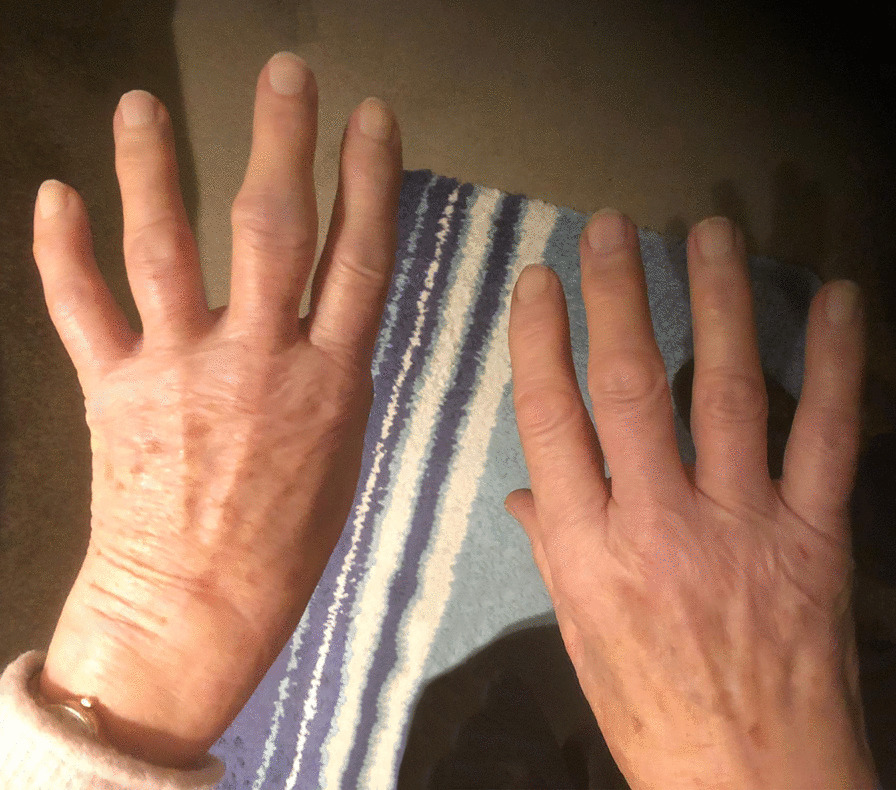
Initial clinical presentation of symmetric edema. Symmetrical edema of the hands 10 days after onset of symptoms. Deformation of the proximal interphalangeal joint of the left middle finger is due to prior existing osteoarthritis

In addition, she reported progressively increasing fatigue over the past 2 months associated with generalized muscle weakness. Pain in the shoulders and hips, as well as neck pain, was also present. The family history for rheumatological diseases was unremarkable. On physical examination, the patient was in a moderately altered general condition. Symmetrical swelling, redness, and localized warmth were noted at the metacarpophalangeal (MCP) and proximal interphalangeal (PIP) joints, as well as pitting edema on the dorsal surface of the hands and wrists (Fig. 2). Her neurological examination was unremarkable, and the rest of the clinical examination was normal.

Laboratory data showed increased C-reactive protein (CRP) to 28 mg/l and erythrocyte sedimentation rate (ESR) to 42 mm/h without leukocytosis. The presence of mild microcytic hypochromic anemia was also noted [hemoglobin (Hb) 103 g/l, hematocrit (Htc) 34%, mean corpuscular volume (MCV) 79 fl, mean corpuscular hemoglobin concentration (MCH) 24.1 pg]. Rheumatoid factor, anti-CCP, myeloperoxidase (MPO), and proteinase 3 (PR3) antibodies were negative. An X-ray of the hands showed only moderate degenerative lesions at the MCP, PIP, and distal interphalangeal (DIP) joints, without lesions (erosions) that could be suggestive of inflammatory arthritis or cartilage calcifications suggestive of chondrocalcinosis. An X-ray of the thorax was normal. Owing to evocative clinical presentation of RS3PE fulfilling the diagnostic criteria, treatment with low-dose glucocorticoids was immediately introduced. Prednisone administration at a dose of 15 mg/day for 2 weeks following a 2-month tapering schedule (decrease of 2.5 mg per week) resulted in an excellent and quick response to treatment with resolution of edemas and pain in less than a week and gradually improving fatigue. The patient remains asymptomatic without treatment, 2 years after the onset of symptoms, without any evident oncologic etiology.

## Discussion

RS3PE was described for the first time in 1985 by McCarty *et al*. [1], but so far the pathological mechanisms remain poorly understood [2–5]. The main diagnostic elements for RS3PE are: (1) pitting edema of the hands and feet, (2) acute onset of polyarthritis, (3) age over 50 years, (4) negative serology for rheumatoid factor [3, 5, 8]. An association with positive human leukocyte antigen (HLA) B7 [1, 3–5] or a high level of vascular endothelial growth factor (VEGF) was observed in several cases, but their role and diagnostic value are debated [2–4, 7, 9].

Several subtypes of RS3PE are described owing to its heterogeneous clinical presentation and potential triggers: idiopathic, drug-induced, or infection-associated forms. Drug-induced forms are associated mainly with the administration of insulin, dipeptidyl peptidase-4 inhibitors, or rifampicin. The infection-induced form seems to be related to parvovirus or *Streptobacillus moniliformis* infection [2, 5, 6, 9]. RS3PE can also coexist with other autoimmune diseases, such as Sjögren’s syndrome, systemic lupus erythematosus (SLE), polyarteritis nodosa, or ankylosing spondylitis [3, 4]. Concerning PMR, some authors suggested that RS3PE is a part of the same spectrum because their clinical presentation can overlap. However, this approach was not confirmed owing to differences summarized in Table 1 [6]. Different classification criteria exist for these two entities (Table 2, Table 3).

**Table 1:** Clinical findings in remitting seronegative symmetrical synovitis with pitting edema, polymyalgia rheumatica, and calcium pyrophosphate (CPP) crystal arthritis [2, 4–6, 10, 11]

Disease	RS3PE	PMR	CPP arthritis
Age (years)	Typically > 50	Over 60	typically > 60
Sex	Male > female	Female > male	=
Symptom onset	Sudden	Progressive	Sudden or progressive
Pitting edema (hands and feet)	Common, symmetrical	−	−
Synovitis small articulations	++	+ (30%)	+
Shoulder and pelvic girdle pain/stiffness	+/−	+++	+
Functional impact	High	Moderate	Moderate
Radiological erosions	−	−	−
Elevation of CRP, ESR	+	++	+
RF	Negative (100%)	Negative/positive (16%)	Negative (100%)
Anti-CCP antibodies	Negative	Negative/positive	Negative
Genetic factors	HLA B7 (55%)	HLA DR4	ANKH gene mutation
Response to corticoid therapy	High, spectacular	High	High
Treatment duration	Short, several weeks/months	2−3 years	Variable
Relapse	Rare	Common within 2 years after diagnosis	Frequent
Paraneoplastic syndrome	20%	2.4%	No
	Up to 30% if association of RS3PE and PMR To be considered if resistant to glucocorticoids		

*CPP* Calcium pyrophosphate, *CRP* C-reactive protein, *ESR* erythrocyte sedimentation rate, *RF* rheumatoid factor, *anti-CCP antibodies* anti-cyclic citrullinated peptides antibodies, *HLA* human leukocyte antigen, *ANKH* the human homolog of the protein product of the murine progressive ankylosis gene. The multipass membrane protein participates in regulating pyrophosphate levels

**Table 2:** Diagnostic criteria of remitting seronegative symmetrical synovitis with pitting edema syndrome [3, 5, 8]

1.	Age over 50 years
2.	Pitting edema in the dorsum of both hands (and feet)
3.	Sudden onset of polyarthritis
4.	Negative serology for rheumatoid factor (RF)

*RF* Rheumatoid factor

**Table 3:** Classification criteria of polymyalgia rheumatica: American College of Rheumatology - European League Against Rheumatism (ACR-EULAR) 2012 [12]

Main criteria		
Age over 50 years		
Bilateral shoulder pain		
Abnormal CRP and/or ESR		
Additional criteria	Without US (points)	With US (points)
Morning stiffness duration > 45 minutes	2	2
Hip pain or limited range of motion	1	1
Absence of RF or anti-CCP antibodies	2	2
Absence of other joint involvement	1	1
At least one shoulder with subdeltoid bursitis and/or biceps tenosynovitis and/or glenohumeral synovitis and at least one hip with synovitis and/or trochanteric bursitis	Not applicable	1
Both shoulders with subdeltoid bursitis, biceps tenosynovitis, or glenohumeral synovitis	Not applicable	1
Diagnosis: all main criteria and	4 or more points	5 or more points

*CRP* C-reactive protein, *ESR* Erythrocyte sedimentation rate, *RF* Rheumatoid factor, *Anti-CCP antibodies* Anti-cyclic citrullinated peptide antibodies

Among the elderly population who present seronegative arthritis without radiological signs of erosive arthritis, which is consistent with our patient’s clinical presentation, several possible differential diagnoses should be considered: RS3PE, late-onset rheumatoid arthritis [LORA, also known as elderly-onset rheumatoid arthritis (EORA)], polymyalgia rheumatica (PMR), and calcium pyrophosphate (CPP) crystal-related arthritis [2, 5, 13].

In most cases of LORA (unlike in the young population), the clinical presentation involves the large proximal joints and not small joints, as in the case of our patient [13]. The absence of RF is not pathognomonic, but the negative antibodies anti-CCP make this diagnosis less likely [3, 5].

CPP crystal-related arthritis can also mimic RA or PMR but is usually associated with specific cartilage calcifications seen on X-rays or in the identification of CPP crystals after joint fluid aspiration [11]. Additionally, a CPP crystal-related edema presents in most patients as asymmetrical and is limited to the involved joint [11].

To distinguish an RS3PE from PMR in our particular case, it is necessary to keep in mind existing diagnostic criteria [1, 6, 10, 13]. Our patient fulfills all criteria for RS3PE (Table 2) based on the clinical features and absence of RF. For PMR, we can apply the ACR-EULAR 2012 classification criteria based on the clinical presentation and optionally the ultrasound findings (Table 3) [12].

In the case of our patient, the main ACR-EULAR criteria for PMR were fulfilled (scapular girdle pain, age over 50 years, increase of CRP and ESR), but additional criteria were not. Additionally, the localization of the morning stiffness was uncommon for PMR and limited to only the hands and feet. Moreover, even if small joint involvement is reported in about 30% of patients with PMR, this does not bring additional points for PMR classification [6].

Consequently, this patient cannot be classified as having PMR according to ACR-EULAR criteria, having only 3 points. PMR diagnosis should be made only after excluding alternative diagnoses [12]. In the case of our patient, the diagnosis of RS3PE was more likely, and the preexisting fatigue could be explained by underlying anemia [1]. However, it is not excluded that shoulder, neck, and pelvic girdle pain could be a part of prodrome symptoms for RS3PE [1].

The differential diagnosis between PMR and RS3PE can be challenging because common clinical symptoms and diagnostic criteria are essentially based on clinical presentation [1, 5–7]. Up to 10% of patients with RS3PE can present as an early form of PMR, or it may occur in its course [7]. Both conditions show good response to low-dose glucocorticoid therapy [1, 3–7, 10], but this element cannot be used as a classification item for PMR according to ACR-EULAR recommendations [12]. The length of therapy for RS3PE is usually significantly shorter than in PMR (2–3 months versus 12–18 months [5, 10]), but the doses of prednisone are similar (15 mg/day at the beginning of therapy following a tapering schedule). A rapid spectacular functional improvement is observed for RS3PE, following edemas’ progressive and complete resolution with no residual sequelae. In both PMR and RS3PE, the presence of malignancy should be actively investigated in case of relapse during glucocorticoid tapering [6, 7, 10]. According to the literature, the risk of malignancy is estimated at about 20% with RS3PE [2, 6] and up to 28% for patients with RS3PE associated with PMR [7].

## Conclusions

Our case suggests that, for some patients with RS3PE, the clinical presentation can be misleading and confounded with early-stage PMR. Using existing classification criteria may help make the final diagnosis and determine the duration of the therapy. It is important to limit, if possible, long-term corticosteroid use, especially for the elderly population, owing to their multiple side effects. Our patient has completed a 2.5-year relapse-free observation period. She did not present any new symptoms, even if the treatment was relatively short (8 weeks). No clinical elements suggest underlying malignancy such as weight loss, night sudation, anemia, or unexplained weakness. No further investigations were made owing to patient personal decision and absence of RS3PE relapse.

## Data Availability

All data generated or analyzed during this study are included in this published article.

## Abbreviations

Anti-CCP antibodies: Anti-cyclic citrullinated peptide antibodies
Anti-MPO antibodies: Anti-myeloperoxidase antibodies
Anti-PR3 antibodies: Anti-proteinase 3 antibodies
CPP: Calcium pyrophosphate
CRP: C-reactive protein
DIP joints: Distal interphalangeal joints
ESR: Erythrocyte sedimentation rate
HLA: Human leukocyte antigen
Hb: Hemoglobin
Htc: Hematocrit
LORA: Late-onset rheumatoid arthritis [also known as elderly-onset rheumatoid arthritis (EORA)]
MCH: Mean corpuscular hemoglobin concentration
MCP: Metacarpophalangeal
MCV: Mean corpuscular volume
PIP: Proximal interphalangeal
PMR: Polymyalgia rheumatica
RA: Rheumatoid arthritis
RF: Rheumatoid factor
RS3PE: Remitting seronegative symmetrical synovitis with pitting edema
SLE: Systemic lupus erythematosus
VEGF: Vascular endothelial growth factor
